# Multidisciplinary Therapy Managed Recurrent Glioblastoma in a BRAF-V600E Mutant Pregnant Female: A Case Report and Review of the Literature

**DOI:** 10.3389/fonc.2020.522816

**Published:** 2020-09-29

**Authors:** Chaoying Qin, Wenyong Long, Chi Zhang, Yuanyang Xie, Changwu Wu, Yang Li, Qun Xiao, Nan Ji, Qing Liu

**Affiliations:** ^1^Department of Neurosurgery in Xiangya Hospital, Central South University, Changsha, China; ^2^Department of Neurosurgery in Beijing Tiantan Hospital, Capital Medical University, Beijing, China

**Keywords:** glioblastoma (GBM), pregnancy, multidisciplinary therapy (MDT), BRAF V600E, vemurafenib

## Abstract

**Background:** Glioblastoma (GBM) is the most malignant intracranial tumor in adults. However, the overall management of GBM in pregnancy is rarely reported. How to balance the therapeutic benefits to the mother and risks to the fetus remains hugely challenging for clinicians. The application of specific targeting therapy combined with conventional treatment sheds light on a longer lifetime for the patients suffering from GBM.

**Case Presentation:** We present a pregnant female at 20 weeks gestation diagnosed with GBM. Surgical resection was initially performed without adjuvant therapy, and the tumor recurred *de novo* 2 months later. A secondary craniotomy and cesarean section were performed simultaneously at 32 weeks gestation, both the patient and infant were survived. She was subsequently treated with traditional chemo-radiotherapy. No other identified genetic alterations indicating an optimistic prognosis were detected except for BRAF V600E mutation. Thus, the BRAF inhibitor was placed on her with achieving a good clinical outcome of more than 2-year survival without recurrence.

**Conclusion:** Personalized multidisciplinary therapy should be considered when GBMs occur in pregnancy. Response to the therapy in this presenting case suggests that BRAF V600E mutation is a favorable biomarker for GBM. The mortality of GBM might be reduced through genetic testing and targeted treatment. However, more studies must be conducted to confirm our observation.

## Background

Glioblastoma (GBM) is the most aggressive brain tumor which is considered a grade IV glioma based on the WHO classification (1), with a median survival of only 3 months in untreated patients (2) and 15 months after conventional therapy (3, 4). Pregnancy diagnosed with GBM is rarely reported; how to balance the therapeutic benefits to the mother, and harmful effects to the fetus is exceptionally challenging for clinicians. The craniotomy is highly risky for both pregnant patients and the fetus. Moreover, radio-chemotherapy is associated with impaired ovarian function, which may impact normal physical development of the embryo (5, 6). To date, the optimal timing of surgery, the utilization of radio-chemotherapy in pregnancy remains deeply controversial (7), and the prognosis of GBM during any stages of pregnancy is extremely poor. Thus, the management of GBM presenting during pregnancy is of great importance to the patient, fetus, family, and clinicians of multidisciplinary teams (8).

With the innovation of genomic characterization, more profound insights into the molecular identity of tumors have been achieved. Increasing molecular targets aiming at the stages of initiation, development, and metastasis of tumors have been identified, through which various novel targeted treatment regimens are created. Multidisciplinary therapies that are composed of targeted therapies, surgery, radiotherapy, and adjuvant chemotherapy have shown efficacy in multiple malignant entities including tumors of the central nervous system (9, 10). The proto-oncogene B-Raf (BRAF) encodes a serine/ threonine-protein kinase of the RAS-RAF-MEK-ERK-MAP kinase pathway. This highly regulated pathway controls cell growth and can be disrupted by BRAF alterations. The majority of BRAF mutations are missense mutations at amino acid position 600 that generate a protein with a glutamic acid (E) residue substituted for the normal valine (V) residue (BRAF V600E). The constitutively activated form of BRAF results in excessive cell proliferation, subsequently promoting tumor growth (11, 12). It has been documented that leukemia (13, 14), colorectal cancer (15), and non-small cell lung cancer (16, 17) contain BRAF mutation. Importantly, successful regression in tumors including melanoma (18) and craniopharyngioma (19) with BRAF inhibitors such as vemurafenib has been reported. The occurrence of BRAF mutation in glioblastoma is rare, which was found in 2 out of 34 (6%) glioblastoma patients (20, 21). A study reported by Ceccon et.al. manifested that BRAF targeted therapy could become an optional approach for GBM patients, especially for those with chemo-resistance or radiotherapy intolerance (18, 22). However, to the best of our knowledge, no data about BRAF inhibitors toward pregnancy with GBM is reported yet.

Here, we present the first case of a young pregnant female with glioblastoma exhibiting BRAF V600E mutation, in whom the isocitrate dehydrogenase (IDH1), O-6-methylguanine-DNA methyltransferase (MGMT) promoter methylation and telomerase reverse transcriptase (TERT) are all negative, indicating chemo-resistance to the classical chemotherapy strategy. The tumor was short-termed recurrent after the primary surgical resection without concomitant treatment. Multidisciplinary therapy composing of mainstream modality for glioma revolved around the second surgery, radiation, chemotherapy and molecular therapy using BRAF V600E inhibitor, was placed on her, resulted in stable regression of the tumor for more than 2 years so far. Besides, the management of GBMs in pregnancy that were reported in the literature were also reviewed and summarized.

## Case Presentation

**Figure 1:**
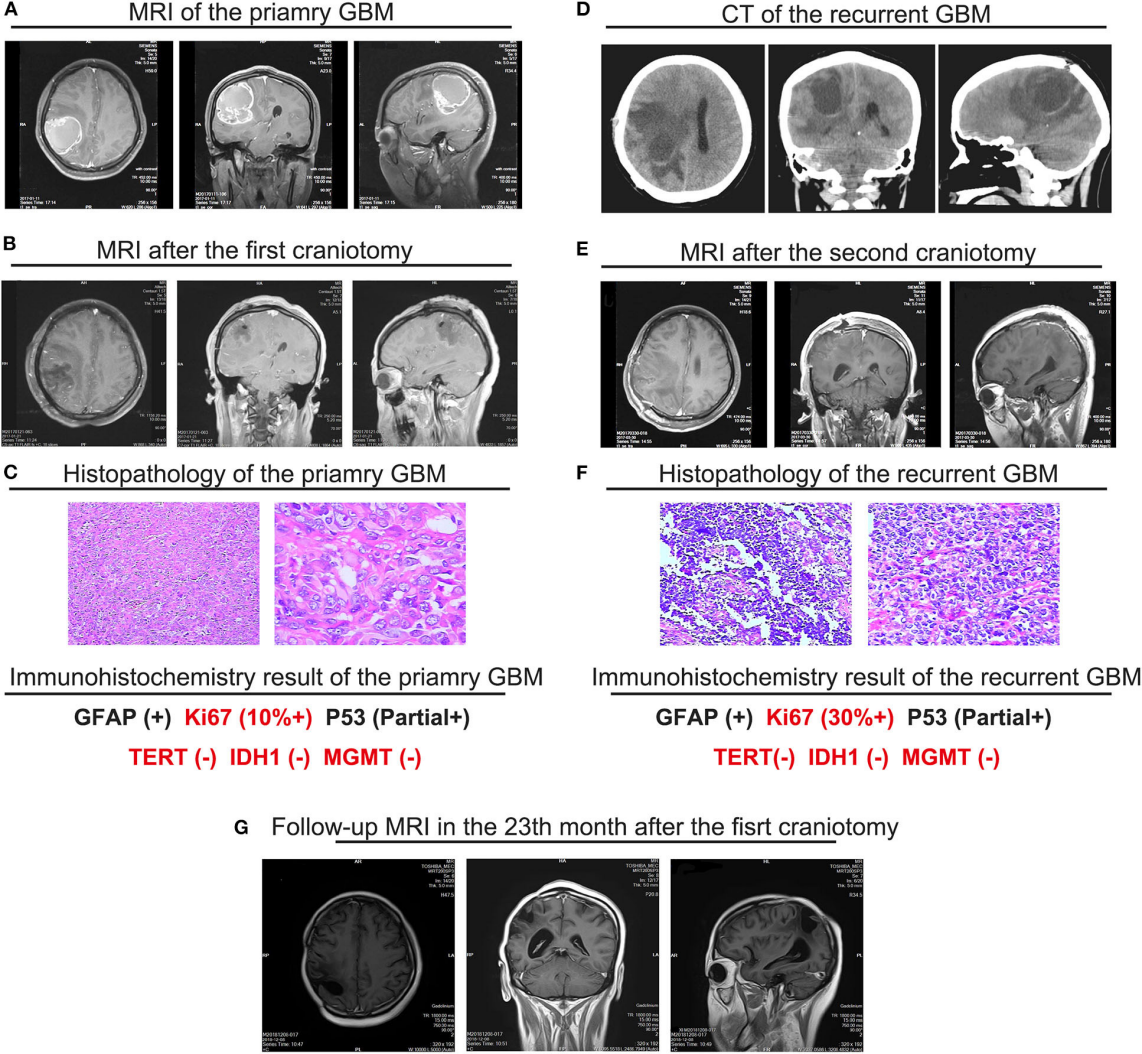
Main medical imaging and histopathology information of the pregnant GBM patient. **(A)** MRI images of the primary GBM prior to the first right parietal craniotomy that demonstrated a heterogeneous cystic and solid mass in the right parietal lobe with marked surrounding edema and a shift of the midline structures to the left side (01/11/2017). **(B)** MRI images on the 3rd day after the first right parietal craniotomy that demonstrated a total resection of the mass, the marked surrounding edema and a shift of the midline structures to the left side remained (01/21/2017). **(C)** Histopathological features of the primary GBM. Hematoxylin and eosin (H & E) staining of the primary revealed the hyper-cellular astrocytic neoplasm. The features such as mitotic activity, microvascular proliferation, and pseudopalisading presented. The histopathological features indicated the primary tumor to be GBM. **(D)** Emergency CT scan when the patient was diagnosed with a recurrent tumor, prior to the second right parietal craniotomy which demonstrated a huge fresh mass of low density in the right parietal lobe surrounded by extensive edema, accompanied with a shift of midline structure and distortion of the right lateral ventricle (03/28/2017). **(E)** MRI images on the 3rd day after the second right parietal craniotomy which demonstrated a total resection of the recurrent tumor, the marked surrounding edema and a shift of the midline structures to the left side still remained (03/30/2018). **(F)** Histopathological features of the recurrent GBM. Hematoxylin and eosin (H & E) staining of the primary revealed the similar histopathological features with the primary tumor. **(G)** MRI images of the latest follow-up after multidisciplinary therapy (12/08/2018). The images demonstrated no signs of recurrence and smaller volume of the surrounding edema. The region of the tumor transformed into a capsule without enhancement. The disease remained stable.

A 28-year old female at 20 weeks' gestation was admitted to our hospital with progressive headache, dizziness, emesis and vision diminution for 2 months. Magnetic resonance imaging (MRI) showed a heterogeneous cystic and solid mass in the right parietal lobe with marked surrounding edema and a shift of the midline structures to the left side; the cyst wall is distinctly enhanced with contrast medium. GBM was highly suspected (Figure 1A). Total resection of the tumor was performed (Figure 1B) with histopathological diagnose of GBM (Figure 1C). Immunohistochemistry exhibited the proliferation marker ki67 was up to 10%, MGMT low methylation, and no IDH1/2 mutation was detected, indicating the tumor was highly aggressive with fairly poor prognosis. With respect to the patient's own will, no radiation therapy or concomitant chemotherapy was applied after surgery.

Unfortunately, over 2 months after the operation (32 weeks gestation), with complaints of dizziness and headache, the patient was transferred to our hospital again. An emergency CT scan supported tumor recurrence (Figure 1D). B-mode ultrasonography showed the fetus was healthy with matured lung. The next day, the patient presented loss of consciousness and right mydriasis all of a sudden. After emergent dehydration treatment, cesarean section and second craniotomy (Figure 1E) were performed contemporarily. Both the mother and infant were survived. Once again, the pathological diagnosis was GBM (Figure 1F), but ki67 was more than 30%, suggesting the recurrent tumor was more aggressive and contained a higher risk of relapse than the primary tumor. Traditional RT and chemotherapy were placed on the patient (Table 2). Considering the short-termed recurrence after the first total resection, Ki67 jumped to 30% + from 10% + in addition, gene sequencing was performed for the patient, which demonstrated no MGMT methylation or IDH mutation (R132H and non-canonical). But BRAF V600E mutation was detected Table 3, showing targeted therapy with BRAF inhibitor may bring benefits for the patient. Thus, vemurafenib was applied (720 mg, twice a day for 28 days), well-toleration except for the diffuse palpable follicular rashes was achieved. Follow-up MRI showed stable disease with no recurrence in the right parietal area (Figure 1G). It has been more than 2 years from the initiation of the multidisciplinary therapy to the latest follow-up examination. The life quality of the patient was satisfied with a healthy baby in the meantime. The regular follow-up information is exhibited as supplementary data (Supplementary Figures 1–4).

### Literature Review

The management of GBMs in pregnancy were diverse and customized, which was highlighted by a literature review of published cases (Table 1). We conducted a PubMed search using glioblastoma/GBM AND (“pregnancy” OR “gestation”) and selected “clinical study” and “case report” as the article type. These search terms yielded 10 previous case reports of GBMs in variant stages of pregnancy and the personalized treatment strategy placed on those cases.

**Table 1:** Defined cases of GBM in pregnancy published in the literature.

**Author/year**	**Age**	**Weeks of gestation**	**Molecular confirmation**	**Treatment strategy**	**Outcome**
Mackenzie et al. (23)	48	36 2/7 (Primary diagnosis of GBM)	NR	• C/S + subsequent craniotomy • RT + adjuvant chemotherapy	• Patient: Died after 9 months since first diagnosis • Infant: remained normally
McGrane et al. (5)	33	14 (Primary diagnosis of GBM)	NR	• Craniotomy • RT + concomitant TMZ chemotherapy (during pregnancy) • C/S • Adjuvant chemotherapy (TMZ)	• Patient: alive without recurrence (2 years FU) • Infant: remained normally
Scarrott et al. (24)	32	24 (recurrent GBM)	NR	• **Primary tumor** (without pregnancy): Craniotomy + RT + concomitant chemotherapy (TMZ) • Got **pregnant** • **Recurrent tumor** (at 24th weeks of) gestration: CS + chemotherapy (procarbazine, vincristine and CCNU, PVC)	• Patient: Died 5 months after delivery • Infant: remained normally
Nolan et al. (25)	23	2 (During adjuvant chemotherapy for primary GBM)	NR	• Craniotomy • RT + concomitant chemotherapy (TMZ) • Got **pregnant** • Continue chemotherapy (TMZ) • Delivery	• Patient: alive (6 months FU) • Infant: remained normally
Flechl et al. (26)	37	3 weeks after the 2nd adjuvant chemotherapy	NR	• **Primary tumor**: 1st Craniotomy + RT + concomitant and adjuvant chemotherapy • **Recurrent tumor**: 2^nd^ Craniotomy + adjuvant chemotherapy (TMZ) • Got **pregnant** • C/S (32 weeks of gestation) • 3rd Craniotomy + chemotherapy (fotemustine and bevacizumab)	• Patient: alive with distal progression (8 months FU) • Infant: NR
Gulsen et al. (27)	30	7 (Primary diagnosis of GBM)	NR	• **Primary tumor**: 1st craniotomy (7th week of gestation, no adjuvant therapy) • **Recurrent tumor**: ■ 2^nd^ craniotomy (24^th^ week of gestation) + RT (27^th^ week of gestation) ■ C/S + 3^rd^ craniotomy (no adjuvant chemotherapy)	• Patient: Died 2 months after delivery • Infant: NR
Horowitz et al. (28)	37	23 (Primary diagnosis of GBM)	MGMT(-)	• Craniotomy • RT (During pregnancy) • Adjuvant chemotherapy (TMZ) (postnatal)	• Patient: alive without recurrence(6 months FU) • Infant: NR
Yust-Katz et al. (29)	NR	NR	NR	• Pregnant when the GBMs were diagnosed • Case 1 • Craniotomy (during pregnancy). No RT/Chemotherapy • C/S • Case 2 • Craniotomy (during pregnancy + RT during pregnancy. No chemotherapy • C/S	• Patient: NR • Infant: healthy
Al-Rasheedy et al. (*9*)	36	18 (During adjuvant chemotherapy for primary GBM)	NR	• RT + concomitant chemotherapy (TMZ) • Adjuvant chemotherapy (TMZ) • C/S (28 weeks of gestation)+ palliative TMZ treatment	• Patient: died 2 weeks after delivery • Infant: remained normally
Taylan et al. (30)	24	24 (When the recurrent tumor was diagnosed)	NR	• Primary tumor: Craniotomy + RT+ concomitant chemotherapy(TMZ) • Got pregnant • Tumor recurred: VP-Shunting+ betamethasone treatment • C/S + planned chemotherapy(TMZ)	• Patient: No follow-up information • Infant: No follow-up information

*NR, Not Recorded; C/S, Cesarean Section; RT, Radiation Therapy; TMZ, Temozolomide; FU, Follow Up; GBM, Glioblastoma*.

**Table 2:** Postoperative Chemo-radiotherapy Strategy.

**Chemotherapy**	**Cycles**	**Volume**	**Date**
Nimotuzumab	concomitant	200 mg/time/week	2017.04.17–2017.05.28
Temozolomide	concomitant	75 mg/m^2^/day	2017.04.17–2017.05.28
Vemurafenib	1 cycle	720 mg/time twice a day	2017.06.09–2017.07.12
Temozolomide (Adjuvant)	1^st^ cycle	300 mg/day	2017.06.27–2017.06.31
	2^nd^ cycle	350mg/day	2017.07.24–2017.07.28
	3^rd^ cycle	350mg/day	2017.10.12–2017.10.16
	4^th^ cycle	350mg/day	2017.11.15–2017.11.19
	5^th^ cycle	300mg/day	2017.12.17–2017.12.21
	6^th^ cycle	300mg/day	2018.01.14–2018.01.18
Radiotherapy	PTV1 60Gy/2.0Gy/30F; PTV2 54Gy/1.8Gy/30F		

**Table 3:** Major Findings of Genetic Variant Analysis by Gene Sequencing.

**Testing gene**	**Result**
MGMT methylation	None
1p Chromosome deletion	Positive
19q Chromosome deletion	None
IDH1 Mutation	None
TERT promoter C228T mutation	None
TERT promoter C250T mutation	None
BRAF V600E mutation	Positive

*IDH1, the isocitrate dehydrogenase-1; MGMT, O-6-methylguanine-DNA methyltransferase; TERT, telomerase reverse transcriptase*.

Some of the pregnant patients chose to get RT and/or chemotherapy after surgical resection by taking a risk for the fetus even at the metaphase of gestation. In the case reported in 2012 (5), the 14-week gravida accepted surgical resection during gestation immediately after the tumor was diagnosed. She decided to undergo RT and concomitant chemotherapy (TMZ) during pregnancy, subsequently delivered the full-term infant and got adjuvant chemotherapy (TMZ) afterward. The patient was alive without progression for 2 years, and the baby was healthy. It was a relatively satisfied outcome for management of GBM in pregnancy. With respect to another case reported in 2014 (28), the 23-week gravida was diagnosed with GBM, the mass was immediately resected. She accepted RT alone during pregnancy and followed postnatal adjuvant chemotherapy (TMZ). The patient was alive without recurrence for 6 months until the case was reported, while there is no information about the infant. It is suggested that undergoing RT, concomitant and adjuvant chemotherapy with taking a risk for the fetus during pregnancy may elevate the survival of the patients, and no evidence of harm to the fetus was reported.

Considering the risk for the fetus, some of the gravidas refused taking RT or chemotherapy after the surgical resection. The patient of 2014 (27) was only 7-weeks pregnant when the tumor was diagnosed and a craniotomy was performed, the family rejected any adjuvant management until the tumor recurred in the 24th week of gestation, the second craniotomy was performed and RT was undergone 3 weeks later. Tumor recurred again in the 37th week of gestation, the cesarean section and third craniotomy were performed contemporarily, neither adjuvant RT nor chemotherapy was given to the patient, this patient died 2 months after delivery. No information of the infant was introduced. The patient in the case report in 2012 (24) got recurrent GBM in the 24th week of gestation, she accepted cesarean section in the 31st week of gestation and postnatal chemotherapy (Procarbazine, Vincristine and CCNU, PVC) was performed. The patient died 5 months after delivery, and the infant was in good condition. Collectively, these cases suggest fearing affecting the development of the fetus, surgical resection alone may lead to a rapid relapse.

In some cases, the patient was diagnosed with GBMs at the relatively late stage of gestation, they chose to undergo delivery and craniotomy in the meantime and accepting adjuvant treatment afterward. The gravida reported in 2005 (23) was almost full-term when she was primarily diagnosed with GBM. She chose to take the risk of undergoing cesarean section (C/S) and craniotomy contemporarily. The concomitant RT and adjuvant chemotherapy were applied, but the patient died after 9 months with the infant remaining normal. In the aforementioned situation, the simultaneous C/S and craniotomy are feasible in specific circumstances and diminishes the potentially harmful effect on the fetus of RT and chemotherapy. However, the risk of performing two operations at the same time is worth considering.

Notably, some patients got pregnant during the period of adjuvant treatment for GBM. The patient reported in 2012 (25) got pregnant during the period of concomitant chemotherapy(TMZ) treatment, the adjuvant TMZ treatment was continuously accomplished following the initial strategy. No abnormality was observed from the infant, and the patient was alive for 6 months when the case was published. The patient reported in 2013 (26) got surgical resection, RT and chemotherapy (TMZ) for the primary GBM, the tumor recurred, and a second surgery was performed followed with adjuvant chemotherapy, however, she got pregnant after complete adjuvant chemotherapy (TMZ) for the recurrent GBM. Cesarean section was performed in full term with a healthy infant being delivered, no treatment aiming the GBM was applied. The tumor progressed, then clinicians performed craniotomy again with chemotherapy (Fotemustine and Bevacizumab) undergoing. The patient was alive with distal progression when the case was reported.

## Discussion

GBM is the most aggressive brain tumor with infiltrating growth, which leads to difficulty of total resection (2, 31–33) and disease progression or recurrence (34). Once recurrence takes place, GBM becomes quickly fatal in the majority of cases (1, 35, 36). Therefore, newly diagnosed GBM requires multidisciplinary approaches to generate the best outcome. GBM during pregnancy is rare in current reports, and the strategy for managing pregnant GBM patients remains largely unknown. The role, safety, and especially the timing of treatment modalities for glioblastoma in the context of pregnancy are personalized for individuals at different stages of pregnancy (7, 23). According to our results and previous studies, we could draw a conclusion that surgical resection is always considered if the tumor is resectable. However, surgery alone results in poorer outcomes of short-termed recurrence compared with multimodal therapy. To date, there is no evidence shows exposure to RT or chemotherapy doing harm to the fetus. Besides, some patients get pregnant during the radiation or chemotherapeutic treatment for GBM causes unexpected difficulties for the regular treatment, contraception is highly recommended for the female GBM patients during the treatment procedure.

**Figure 2:**
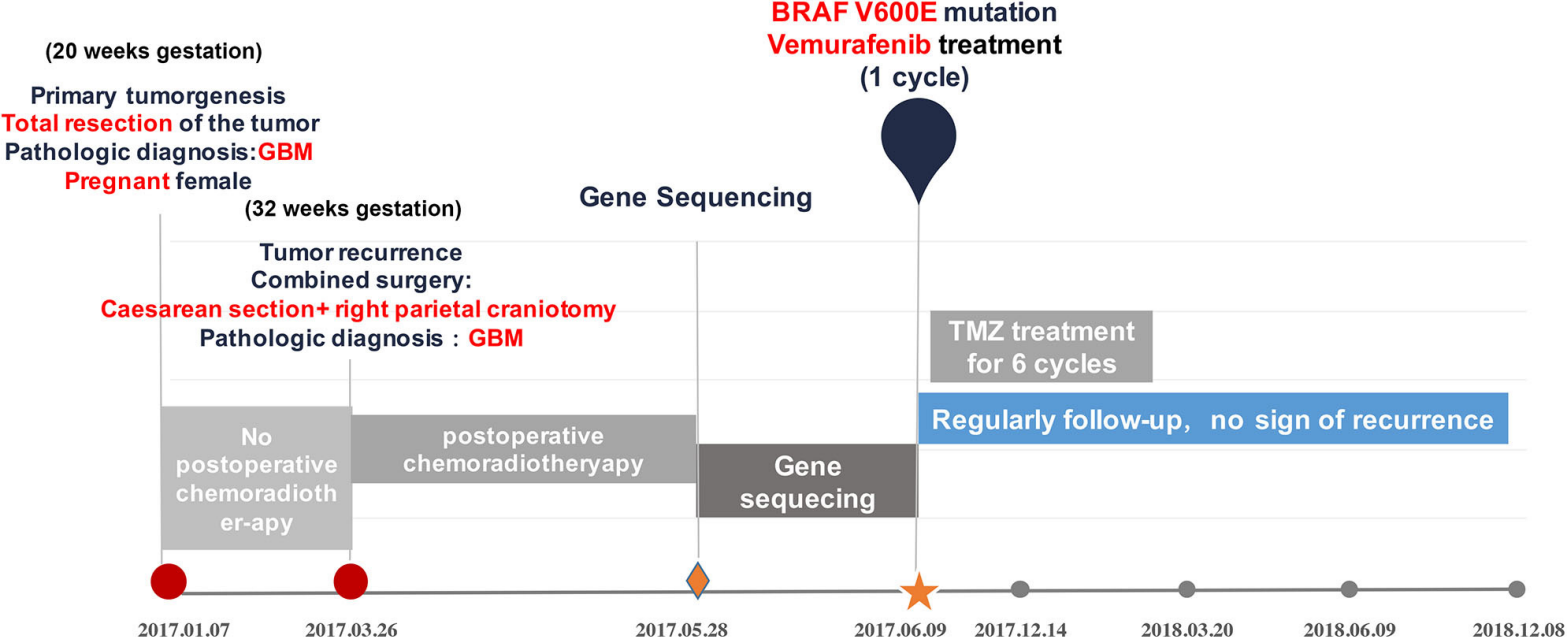
Overview of the patient's course of disease, treatment regimen and genetic analysis.

The reported outcomes of variant management for GBM during pregnancy are dramatically different. Survivals of the mother range from 2 to 24 months, none of the cases accepted gene sequencing for molecular features of the tumors and genetic therapy. Regarding this presenting case, not willing taking a risk for the fetus, the pregnant patient accepted surgical resection for the primary tumor without undergoing any concomitant radiation therapy or adjuvant chemotherapy, consequently, the tumor recurred in a short time. With undergoing multidisciplinary therapy including gene sequencing and BRAF-targeted treatment (Figure 2), she remained stable for 24 months since the tumor was diagnosed primarily even with recurrence. With regard to this case, the pregnant GBM patients obtained satisfactory lifetime with delivering healthy baby meanwhile. Although recent report declared pregnant GBM patients with 64 and 96 months of progression-free survival. The patient with 96 months was IDH1 mutant and the other patient with 64 months had MGMT methylation (37), which are both indicators of optimistic prognosis. To the best of our knowledge, this case is still the maximum lifetime for the pregnant GBM patients without IDH mutation and MGMT methylation. Therefore, this case encourages discussion on the most appropriate adjuvant therapy for pregnant GBM patients who have significant post-operative tumor residuum or progression, in addition, point out the importance of advanced genetic therapy.

Nowadays, more in-depth insights into genetic advances have contributed to a better understanding of the pathophysiology and molecular stratification of GBM (38, 39). The molecular alterations in GBM are extremely complicated. The mostly focused clinically relevant genetic alterations in GBMs include IDH mutations, 1p/19q co-deletion, epidermal growth factor receptor (EGFR) amplification, O-6-methylguanine-DNA methyltransferase(MGMT) methylation status and telomerase reverse transcriptase(TERT) promoter mutation (36, 40–42). Our presenting case showed no alteration in those genes, and MGMT methylation was identified as negative, indicating the traditional chemotherapy and targeting therapy would not undergo well. Moreover, Ki67 of the recurred GBM elevated to 30%+ from 10%+, which suggested the recurrent tumor possessing higher aggressive features compared with the primary one. Fortunately, the gene sequencing technique with wide coverage provided us with another potential approach for stabilizing the progression of the disease since BRAF V600E mutation was detected in the recurrent tumor. The nimotuzamab was administered to reduce the edema of the surrounding area of the postoperative tumor cavity, and also to elongate the period of stable disease. As previously reported, nimotuzumab in addition to standard treatment is well-tolerable and has increased survival in GBM patients with EGFR positive (43), we underwent nimotuzamab with temozolomide and RT contemporarily after the second craniotomy without the gene sequencing result coming out, even though the genetic test claimed EGFR negative in this patient afterwards.

With increasing prominence, BRAF-targeted therapies have shown efficacy in specific tumor entities (11, 12, 44). Direct targeting with BRAF inhibitors has been reported to be favorable for treating those malignant tumors harboring the BRAF V600E mutation (45, 46). In the view of central nervous system, the most commonly reported brain tumors with BRAF mutation include pleomorphic xanthoastrocytoma (PXA) (60%), extra-cerebellar pilocytic astrocytoma (20%) and ganglioglioma (20% to 60%) for low grade (WHO I-II), PXA with anaplastic features (60%) (21, 47–49) for higher grade (WHO III). Several rare CNS tumors such as craniopharyngioma (19, 50) and meningioma (51)were also reported containing BRAF mutation, following encouraging outcomes. As to high-grade brain tumors, recent reports about the GBM (48, 52, 53) and gliosarcoma (54) that manifest BRAF V600E mutation have been published. Of note, the outcomes of the GBMs with BRAF V600E that were treated with vemurafenib are optimistic according to the reports so far.

Meanwhile, the side-effect of vemurafenib is also reported in the clinical cases, for example, granulomatous hepatitis (55) and cutaneous diseases (56). Accordingly, prior to the targeting therapy with vemurafenib for our patient, hepatic protection was undergone. Severe diffuse palpable follicular rashes presented without prediction, so the treatment had to be halted for 5 days, and the patient was recommended to wear protective clothing and take other measures such as using sunscreens containing UVA-protective agents to prevent photosensitivity. However, the vemurafenib was administered for only 1 cycle due to the iterative the diffuse palpable follicular rash afterwards, the patient complained the adverse event was intolerable and refused to accept the following treatment. The patient and her family were not able to afford the high cost of subsequent vemurafenib treat. Despite the side-effect, BRAF V600E mutation can be regarded as a potential hallmark indicating a relatively optimistic prognosis for the patients suffering from GBM because BRAF inhibitors such as vemurafenib extended the survival of GBM patients. We propose that BRAF mutation should be tested routinely for GBMs and the other malignant gliomas during the histopathological assay. Besides, with the revolution of genomics, gene sequencing has become a portion of multidisciplinary therapy, which would provide the physicians with adequate information guiding the therapeutic strategy. The novel targets such as BRAF might shed light on the pessimistic prognosis of GBM. However, further observation and potential adjustment is required to determine the optimal duration, dose, and combination of multidisciplinary treatment. On the other hand, the influence of targeted therapy on the maturity and development of the fetus is largely unknown due to the lack of reported cases.

## Conclusion

We present here a significant case that suggests the personalized MDT comprising genetic therapy may provide the pregnant GBM patients with improved outcomes, it complements the evidence of the management for GBMs in pregnancy. The response to this presenting therapy indicates that BRAF V600E mutation is a favorable biomarker for GBM. The mortality of GBM might be reduced through genetic testing and vemurafenib targeted treatment. However, more studies must be conducted to confirm our observation.

## Data Availability Statement

The raw data supporting the conclusions of this article will be made available by the authors, without undue reservation, to any qualified researcher.

## Ethics Statement

The studies involving human participants were reviewed and approved by Ethics Committee of Xiangya Hospital Central South University. The patients/participants provided their written informed consent to participate in this study. Written informed consent was obtained from the individual(s) for the publication of any potentially identifiable images or data included in this article.

## Author Contributions

CQ, WL, YX, CZ, CW, YL, QX, and QL performed the surgical procedures. CQ, WL, CW, YL, and QX performed data collection and analysis. NJ and YL performed gene sequencing and target therapy. CQ, WL, and QL wrote the manuscript. QL supervised the entire work. CQ, WL, YX, CZ, CW, YL, QX, NJ, YL, and QL provided final approval for the version to be published. All authors contributed to the article and approved the submitted version.

## Conflict of Interest

The authors declare that the research was conducted in the absence of any commercial or financial relationships that could be construed as a potential conflict of interest.

## Supplementary Material

Supplementary Figure 1 MRI images prior to vemurafenib therapy (the radiotherapy and concomitant chemotherapy had been accomplished) (05/27/2017). **(A,B)** Axial MRI T1-weighted images with gadolinium-based contrast demonstrated no signs of recurrence, the surrounding edema subsided and the midline structures returned to the initial position. **(C,F)** Coronal and sagittal MRI T1-weighted images with gadolinium-based contrast demonstrated the consistent outcome. **(D,E)** Axial MRI T2-weighted images demonstrated edema around the tumor relived.

Supplementary Figure 2 First regular follow-up MRI images after the multidisciplinary therapy (12/14/2017). **(A,B)** Axial MRI T1-weighted images with gadolinium-based contrast demonstrated no signs of recurrence, the volume of surrounding edema decreased. The region of the primary and recurrent tumor gradually transformed into a capsule without enhancement. **(C–F)** Coronal and sagittal MRI T1-weighted images with gadolinium-based contrast demonstrated the consistent outcome.

Supplementary Figure 3 Second regular follow-up MRI images after multidisciplinary therapy (03/20/2018). **(A,B)** Axial MRI T1-weighted images with gadolinium-based contrast demonstrated no signs of recurrence, the volume of surrounding edema further decreased. The region of the primary and recurrent tumor gradually transformed into a capsule without enhancement. **(C–F)** Coronal and sagittal MRI T1-weighted images with gadolinium-based contrast demonstrated the consistent outcome.

Supplementary Figure 4 Third regular follow-up MRI images of the latest follow-up after multidisciplinary therapy (06/09/2018). **(A,B)** Axial MRI T1-weighted images with gadolinium-based contrast demonstrated similar features with the second former follow-up results. **(C–F)** Coronal and sagittal MRI T1-weighted images with gadolinium-based contrast demonstrated the consistent outcome.

## Abbreviations

MDT: multidisciplinary therapy
GBM: glioblastoma
WHO: world health organization
TMZ: temozolomide
IDH1: the isocitrate dehydrogenase-1
MGMT: O-6-methylguanine-DNA methyltransferase
TERT: telomerase reverse transcriptase
RT: radiation therapy
C/S: cesarean section
EGFR: epidermal growth factor receptor
PXA: pleomorphic xanthoastrocytoma.
