# Supplementary figures and images for: Multidisciplinary Therapy Managed Recurrent Glioblastoma in a BRAF-V600E Mutant Pregnant Female: A Case Report and Review of the Literature
Source: Front Oncol. 2020 Sep 29;10:522816. doi: 10.3389/fonc.2020.522816 (PMC7550879; doi:10.3389/fonc.2020.522816)

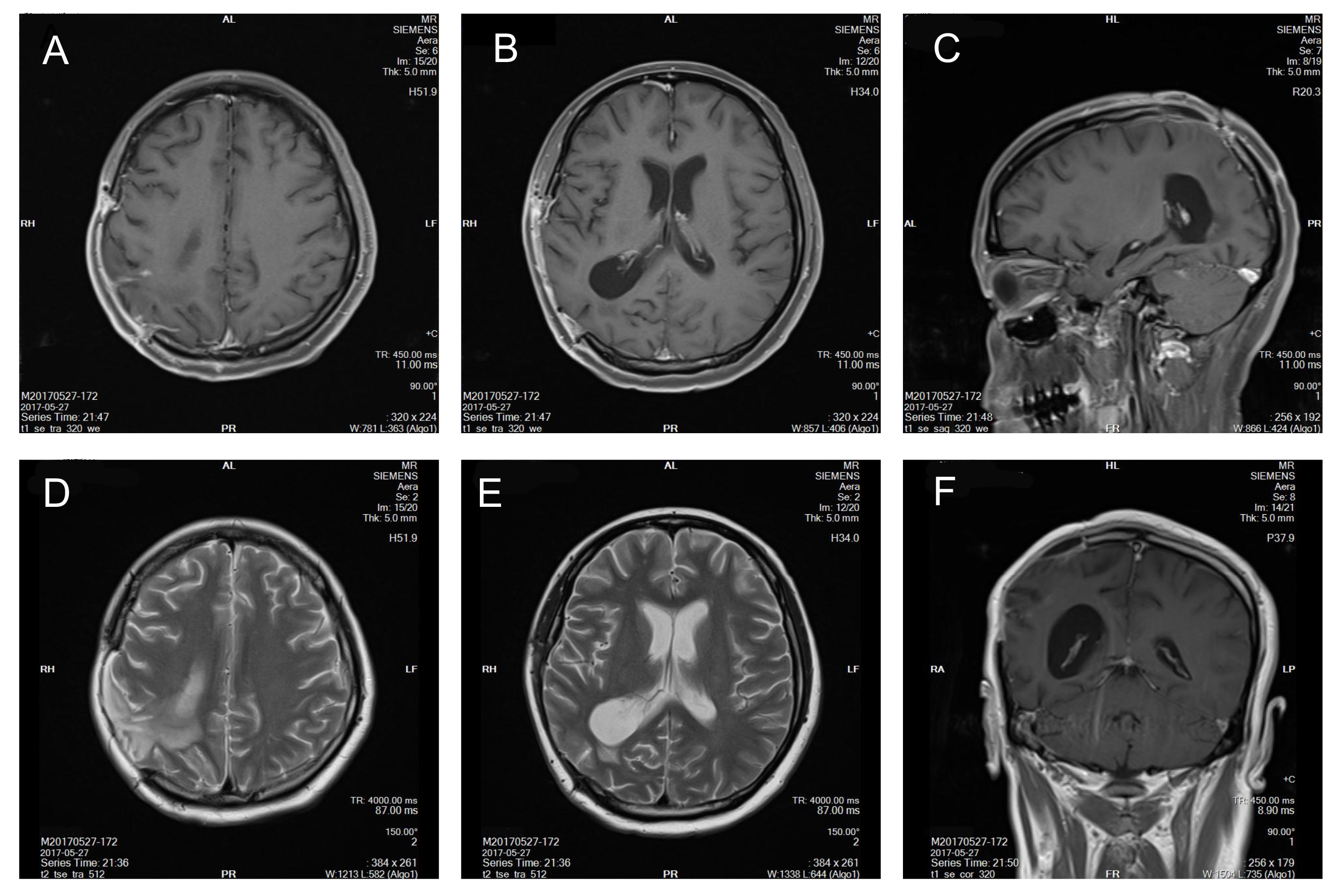

Supplement: Supplementary Figure 1 — MRI images prior to vemurafenib therapy (the radiotherapy and concomitant chemotherapy had been accomplished) (05/27/2017). (A,B) Axial MRI T1-weighted images with gadolinium-based contrast demonstrated no signs of recurrence, the surrounding edema subsided and the midline structures returned to the initial position. (C,F) Coronal and sagittal MRI T1-weighted images with gadolinium-based contrast demonstrated the consistent outcome. (D,E) Axial MRI T2-weighted images demonstrated edema around the tumor relived. [file Image_1.TIF]

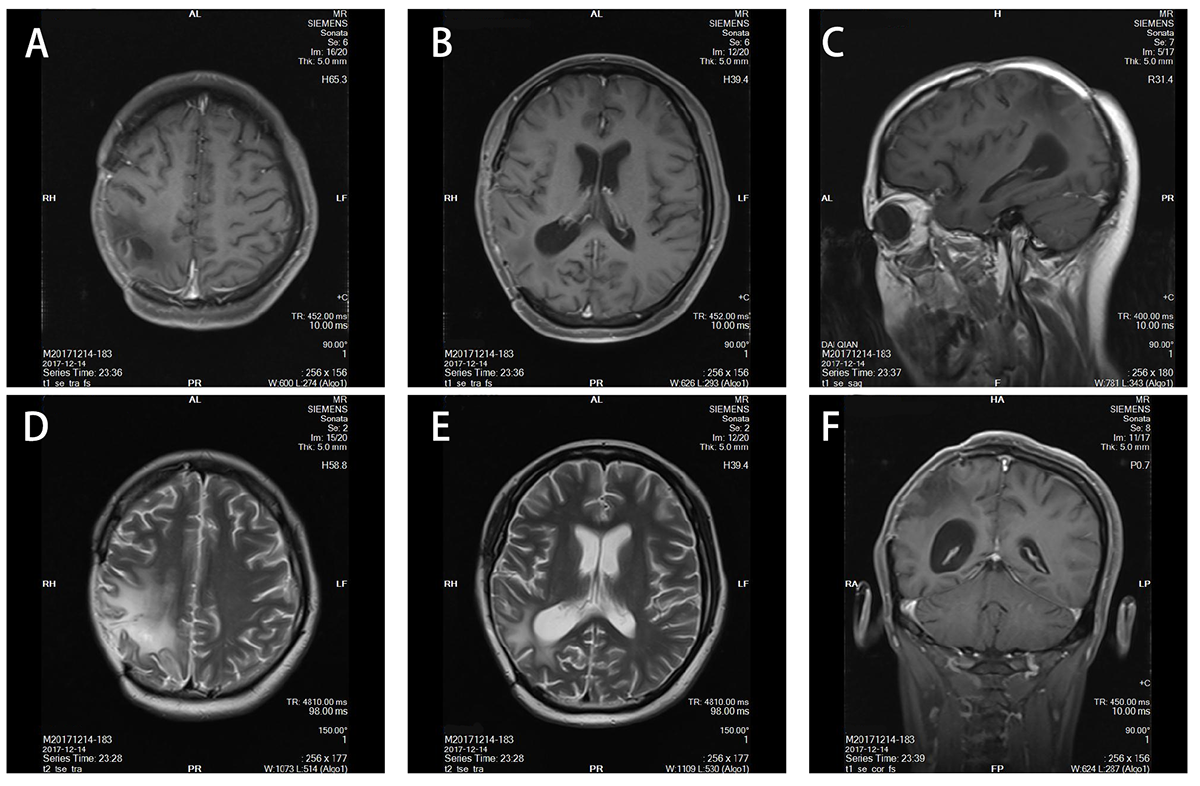

Supplement: Supplementary Figure 2 — First regular follow-up MRI images after the multidisciplinary therapy (12/14/2017). (A,B) Axial MRI T1-weighted images with gadolinium-based contrast demonstrated no signs of recurrence, the volume of surrounding edema decreased. The region of the primary and recurrent tumor gradually transformed into a capsule without enhancement. (C–F) Coronal and sagittal MRI T1-weighted images with gadolinium-based contrast demonstrated the consistent outcome. [file Image_2.TIF]

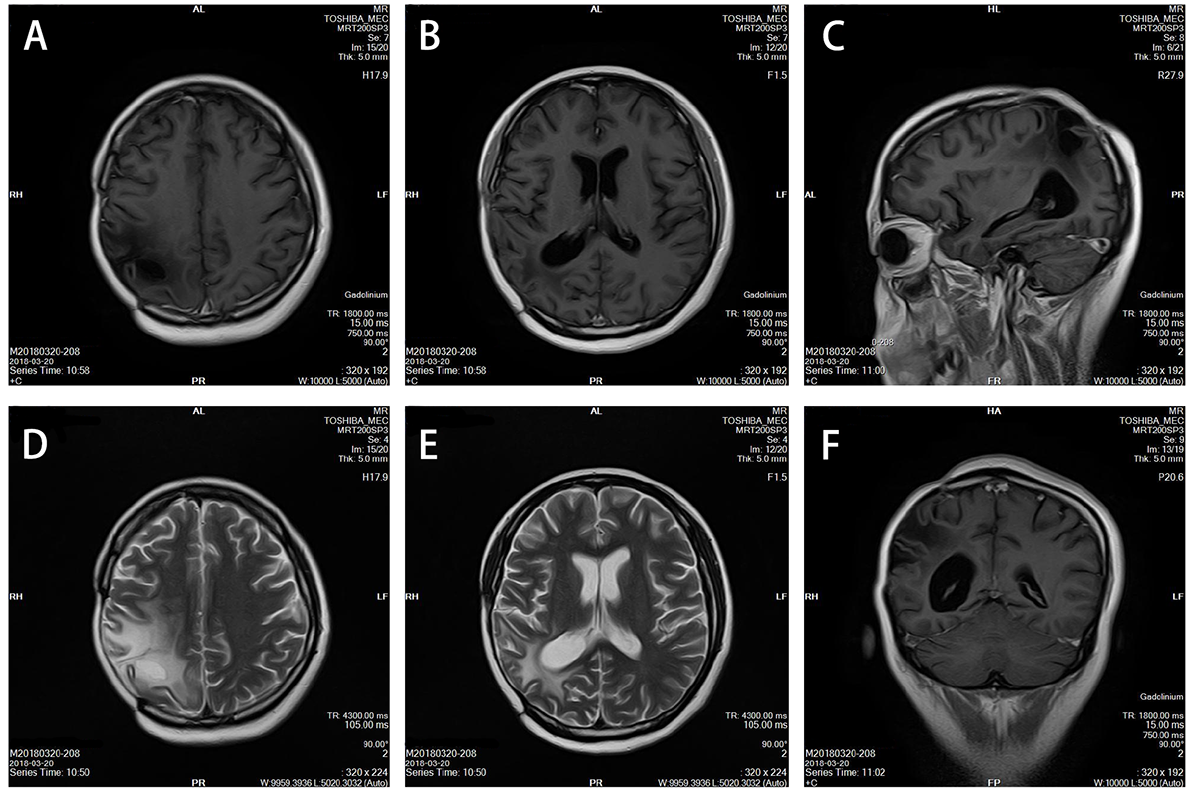

Supplement: Supplementary Figure 3 — Second regular follow-up MRI images after multidisciplinary therapy (03/20/2018). (A,B) Axial MRI T1-weighted images with gadolinium-based contrast demonstrated no signs of recurrence, the volume of surrounding edema further decreased. The region of the primary and recurrent tumor gradually transformed into a capsule without enhancement. (C–F) Coronal and sagittal MRI T1-weighted images with gadolinium-based contrast demonstrated the consistent outcome. [file Image_3.TIF]

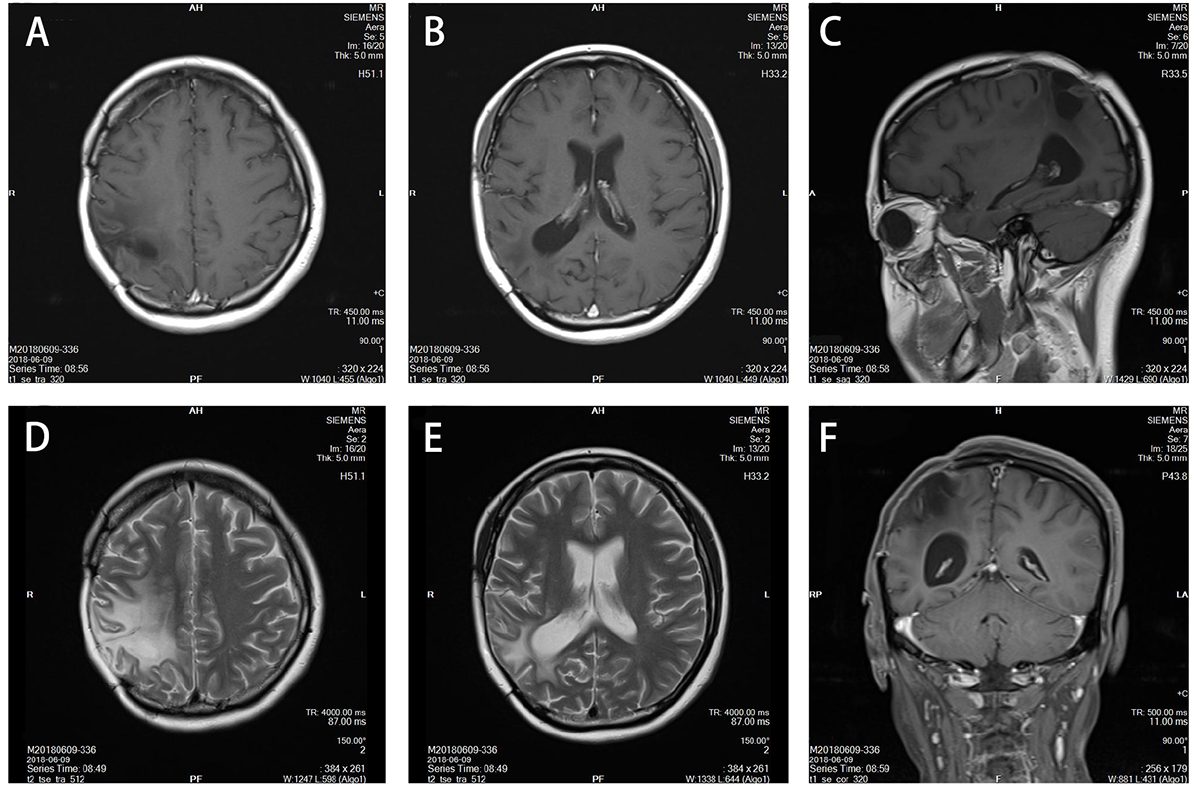

Supplement: Supplementary Figure 4 — Third regular follow-up MRI images of the latest follow-up after multidisciplinary therapy (06/09/2018). (A,B) Axial MRI T1-weighted images with gadolinium-based contrast demonstrated similar features with the second former follow-up results. (C–F) Coronal and sagittal MRI T1-weighted images with gadolinium-based contrast demonstrated the consistent outcome. [file Image_4.TIF]
